# The potential role of integrin alpha 6 in human mesenchymal stem cells

**DOI:** 10.3389/fgene.2022.968228

**Published:** 2022-09-16

**Authors:** Mohammed Al-Obaide, Albi Ishmakej, Christina Brown, Matteo Mazzella, Patrina Agosta, Mick Perez-Cruet, G. Rasul Chaudhry

**Affiliations:** ^1^ Department of Biological Sciences, Oakland University, Rochester, MI, United States; ^2^ OU-WB Institute for Stem Cell and Regenerative Medicine, Oakland University, Rochester, MI, United States; ^3^ Ascension Providence Hospital, Southfield, MI, United States; ^4^ Department of Neurosurgery, Beaumont Health, Royal Oak, MI, United States

**Keywords:** integrin alpha 6, integrin alpha 6-antisense 1, lncRNA, mesenchymal stem cells, self-renewal, promoters

## Abstract

Human mesenchymal stem cells (MSCs) are isolated from various adult and perinatal tissues. Although mesenchymal stem cells from multiple sources exhibit similar morphology and cell surface markers, they differ in their properties. In this study, we determined that the expression of integrin alpha 6 (*ITGA6*) and *ITGA6* antisense RNA (*ITGA6-AS1*) correlates with the proliferation, cell size, and differentiation potential. The expression of ITGA6 was inversely correlated with *ITGA6-AS1* in MSCs. The expression of *ITGA6* was higher, but *ITGA6-AS1* was lower in MSCs from cord placenta junction, cord tissue, and Wharton’s jelly. In contrast, *ITGA6* expression was lower, while *ITGA6-AS1* was higher in MSCs from the placenta. The bioinformatic analysis showed that *ITGA6* genomic DNA transcribes *ITGA6-AS1* from the reverse strand, overlapping *ITGA6* exon-2. Additionally, we identify several putative promoters (P1-P10) of *ITGA6*. *ITGA6-P10* is CG rich and contains CGI. EMBOSS Cpgplot software revealed a CGI length of 180 bp that extends from nucleotide 125 to 304 of the P10 sequence. We suggest that the post-transcriptional regulation of the *ITGA6* in mesenchymal stem cells is controlled by the *ITGA6-AS1,* which could be a critical factor responsible for the heterogeneity in function and cell fate of human MSCs. These results may provide further impetus for investigations to unravel the mechanisms of *ITGA6* regulation that could help maintain or improve the properties of mesenchymal stem cells.

## Introduction

Human mesenchymal stem cells (MSCs) display promising therapeutic properties including propensity to differentiate into multilineage, the ability to home to the site of damage, induce immunomodulatory and anti-inflammatory responses, and are heterogeneous cells that provide neuroprotection (Beeravolu et al., 2016; Beeravolu et al., 2017; Brown et al., 2021). Although MSCs from multiple sources exhibit similar morphology and cell surface markers, they display heterogeneity in features depending upon the source, method of isolation, culture media, and mechanical cues (Kolf et al., 2007; Chen et al., 2015; Elahi et al., 2016; Costa et al., 2021). The culture conditions, intracellular matrix (ICM), and extracellular matrix (ECM) of MSCs can influence genetic-epigenetic inheritance (Maurer, 2011; Sullivan et al., 2014; Assis-Ribas et al., 2018; Matta et al., 2019). Genetic-epigenetic can also influence the MSC’s fate; differentiation into progenitors and terminally differentiated cells (Teven et al., 2011; Cakouros and Gronthos, 2020; Nieto-Nicolau et al., 2020).

The long non-coding RNA (lncRNA) plays an important role in the post-transcriptional regulation of genes. They are the host of microRNAs (miRNAs) produced from non-coding RNAs forming an imperfect stem-loop secondary structure (Ballantyne et al., 2016; He et al., 2019; Zhang et al., 2019; Sun et al., 2021). lncRNA inactivates or destabilizes mRNA by pairing at a specific complementarity sequence or generating a miRNA that targets the mRNA (Zealy et al., 2018; Zhang et al., 2019; Ma et al., 2021; Statello et al., 2021). Antisense RNAs are lncRNAs subtype (Sun et al., 2021) that can generate diverse transcripts, play multifunctional roles including embryonic pluripotency, differentiation and development, are widespread in humans and other eukaryotes (Villegas and Zaphiropoulos, 2015). lncRNAs are also reported to be functional regulators of MSCs (Ju et al., 2019; Li et al., 2019; Xie et al., 2019).

An important group of proteins, the integrin family that includes integrins alpha (ITGA), and beta proteins, play a critical role in the MSCs fate. They are cell surface adhesion receptors that support signaling across the plasma membrane in both ICM and ECM and mediate the intracellular signals response to the ECM (Hynes, 2002). Further, integrins activate a wide range of signaling pathways and are considered the main factor involved in regulating cell growth and mobility, cellular shape, survival, and differentiation associated with ECM interaction (Akiyama, 1996; Yamada, 1997; Chen and Sheppard, 2007; Schwartz, 2010; Becerra-Bayona et al., 2018; Peterson and Koval, 2021). Thus, integrins are a prime link between the MSCs’ ICM and ECM.

Among the ITGAs, *ITGA6*, also known as CD49f, is a transmembrane glycoprotein adhesion receptor protein which show higher expression in bone marrow (BM) MSCs (Nieto-Nicolau et al., 2020). Downregulation of *ITGA6* impaired the cell proliferation and migration of BM-MSCs via the protein kinase B (AKT) pathway and the cell cycle inhibitor proteins p53 and p21 (Nieto-Nicolau et al., 2020). Additionally, overexpression of *ITGA6* was found to be associated with an osteoporotic vertebral fracture in elderly women (Jales Neto et al., 2020), bone metastasis and ductal carcinoma (Martin and Jiang, 2014), colon cancer-initiating cells (Haraguchi et al., 2013), cervical squamous cell carcinoma (Hou et al., 2015), and in the invasion, metastasis and poor prognosis in human gallbladder carcinoma (Zhang et al., 2016).

Our study showed a correlation between the expression of *ITGA6* and *ITGA6* antisense RNA1 (AS1), also known as AC078883.3, is inversely proportional to the properties of MSCs isolated from various sources. The *ITGA6* genomic DNA expresses *ITGA6-AS1* from the reverse strand. Our bioinformatic analysis identified several putative promoters (P1-P10) of *ITGA6*, and *ITGA6-P10* is CG-rich and contains CGI of 180 bp that extends from nucleotide 125 to 304. Our results and bioinformatic analysis indicate that post-transcriptional regulation of the *ITGA6* in MSCs may be controlled by the *ITGA6-AS1*, which could be a critical factor responsible for the heterogeneity in the cell function and fate of human MSCs. These results may provide further impetus for investigations to unravel the mechanisms of *ITGA6* regulation that could help maintain or improve the properties of MSCs.

## Materials and methods

### Human mesenchymal stem cell cultures

In this study, we used well-characterized MSCs isolated from six sources, BM, chorion (CH), cord placenta junction (CPJ), cord tissue (CT), Wharton’s jelly (WJ), and placenta (PC) as described previously (Beeravolu et al., 2016; Beeravolu et al., 2017). All cells were grown in a growth medium (GM) containing DMEM nutrient mix F12 medium (DMEM/F12; Life Technologies, Carlsbad, CA, United States), supplemented with 10% fetal bovine serum (FBS; VWR, Radnor, PA, United States), and 5.6% of antibiotic solution (0.1% gentamicin, 0.2% streptomycin, and 0.12% penicillin) (Sigma Aldrich, St Louis, MO, United States) in a humidified 5% CO2 atmosphere at 37 °C. All cells were grown to 70% confluency at passage 6 (P6).

Determination of Cell size, Doubling Time, and Colony Forming Efficiency of MSCs.

MSC size was measured by using microscopy and ImageJ. The doubling time (DT) and colony-forming efficiency of MSCs were determined as described previously (Beeravolu et al., 2016; Beeravolu et al., 2017).

RNA Extraction and Analysis of *ITGA6* and *ITGA6-AS1* Expression.

Total RNA was extracted from 70% confluent cells using the GeneJET RNA purification kit (ThermoFisher Scientific) following the manufacturer’s instructions. RNA was treated with DNase and incubated at 37 °C for 30 min in the thermocycler (Bio-Rad, Hercules, CA, United States). cDNA was synthesized using iScript kit (Bio-Rad), and RT-qPCR was performed by using SsoAdvanced universal SYBR Green Supermix Kit (Bio-Rad) on CFX96 Real-Time System (Bio-Rad). A 10 µL reaction was used, which included 5 µL SYBR green, 3 µL of distilled water, 0.5 µL of forward primer, 0.5 µL of reverse primer, and 1 µL of 1:10 diluted cDNA. Each reaction was exposed to the following conditions: 98 °C for 10 min, followed by 30 s of 98°C, 20 s of 60°C, and 30 s of 72 C for 44 cycles in 96-well optical reaction plates (Bio-Rad). Human gene GAPDH was used to normalize fold gene expression. Primer sets for *ITGA6*, *ITGA6-AS1,* and GAPDH are listed in Supplementary Table S1. The 2^−∆∆Cq^ method was used to analyze relative gene expression (fold change) data obtained by real-time quantitative PCR (Schmittgen and Livak, 2008).

### Bioinformatic analysis and genomic databases

The URLs of genomics databases and bioinformatics tools used in this study are shown in Supplementary Table S2. The genomic features of *ITGA6* and *ITGA6-*AS1 loci are searched using online public genomics databases NCBI-Gene, UCSC Genome Browser, Ensembl Genome Browser, EMBL-EBI, and Eukaryotic promoter database. EMBOSS Matcher was used to identify local similarities in two input sequences. EMBOSS Needle was used for optimal global sequence alignment and EMBOSS Cpgplot to locate and plot CpG islands (CGI) in nucleotide sequence(s). The miRBase search tool to discover mature microRNA (miR) sequences from long non-coding RNA *ITGA6* antisense RNA 1 (*ITGA6-AS1*), NCBI Reference Sequence: NR_1,57,573.1. Identification of the miR recognition elements (MREs) in the *ITGA6* mRNA, NM001079818.3, was performed by RNA22 v2 miR target detection tool to find miR that binds to the target *ITGA6* mRNA MRE. The UCSC genome browser “get DNA” and “Blat” tools were used to retrieve and analyze sequences in the forward or reverse strands and recover the identified sequences from earlier versions to the updated GRCh38/hg38 version.

### Statistical analysis

The statistical tests, *t*-test, and one-way ANOVA were performed using Excel Data Analysis Toolpack and GraphPad Prism 7.01 (GraphPad Software, Inc., La Jolla, CA, United States) for various parameters. A significant difference was assessed at *p* < 0.05.

## Results

### Properties of mesenchymal stem cells from various sources

**FIGURE 1:**
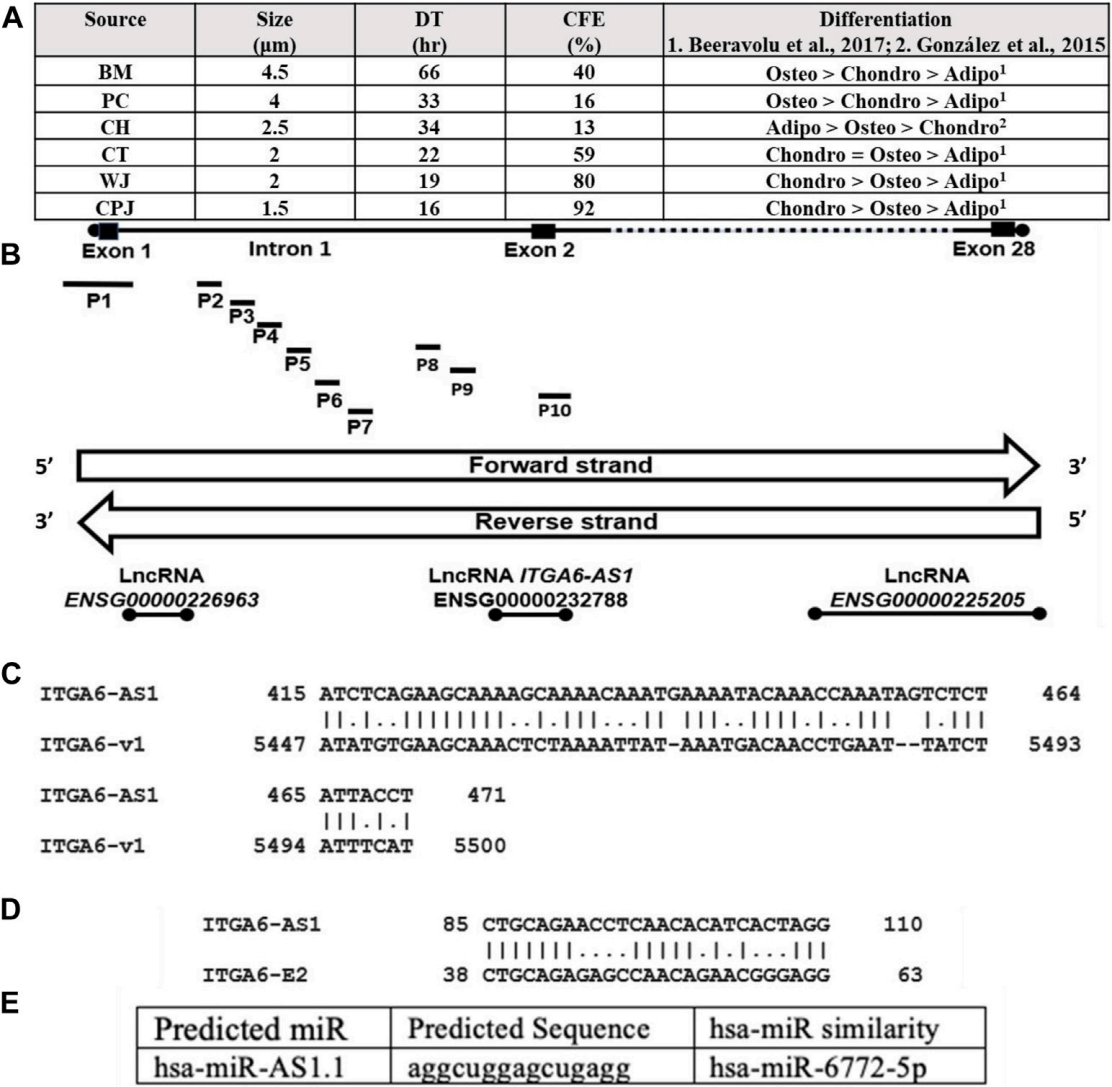
**(A)** Characteristics including cell size, doubling time, CFE, and differentiation potential of human MSCs from six sources, BM. PC, CH, CT, WJ, and CPJ. The *ITGA6* is mapped at chr2:172,427,336–172,506,459 forward strand. **(B)** *ITGA6* genomic DNA hosts three lncRNA loci and ten promoters (P1 to P10) and the lncRNA are coded by the reverse strand (Data from NCBI-Gene and Ensembl databases). **(C)** The *ITGA6-AS1* partial complementarity with 3′UTR of *ITGA6* mRNA type 1/variant 1 (v1). **(D)** *ITGA6-AS1* showed 65.4% alignment to 26 bp of *ITGA6* exon 2 (E2). **(E)** Predicted miR sequence hsa-miR-AS1.1, for details, see Supplementary Table S6.

MSCs isolated from various sources expressed specific cell surface markers including CD29, CD44, CD73, CD90, CD105 as determined by flow cytometry (Brown et al., 2019). All MSCs (P6) were cultured to 70% confluency and analyzed for various properties (Figure 1A). A cell size comparison showed that MSCs from BM, PC, CH, CT, WJ, and CPJ had sizes of 4.5, 4, 2.5, 2, 2, and 1.5 μm, respectively. BM MSCs had the largest, and CPJ had the smallest cell size. The growth rate determined based on the DT correlated with the size of MSCs. The BM cells had the highest, and CPJ had the lowest DT. We also analyzed the clonogenicity of MSCs from various sources. The results showed that MSCs had CFE of 13, 16, 40, 59, 80, and 92% from CH, PC, BM, CT, WJ, and CPJ, respectively. CPJ MSCs had the highest, and CH had the lowest CFE. We have previously shown that MSCs from BM and PC favored differentiation toward the osteogenic lineage more but less toward adipogenic lineage (Beeravolu et al., 2017). MSCs from CPJ and WJ showed increased differentiation towards chondrogenic lineage. CT MSC had a similar propensity to differentiate between chondrogenic and osteogenic lineages. CPJ, WJ and CT had a lower differentiation potential toward the adipogenic lineage. CH MSCs displayed increased adipogenic differentiation but decreased chondrogenic differentiation potential (González et al., 2015). CPJ and WJ displayed increased differentiation towards the chondrogenic lineage. CH MSCs had the lowest clonogenicity potential. BM and PC MSCs with the greater tendency to differentiate into osteogenic lineage were larger in cell size and doubling time. CPJ cells had the smallest cell size, doubling time, and largest CFE compared to the MSCs from other sources. Our published results showed that ITGA6 expression varied significantly among the MSCs from BM, CH, PC, CT, WJ, and CPJ (Brown et al., 2019). ITGA6 expression was highest in CPJ and lowest in both BM and PC. Our results showed a clear correlation between the ITGA6 expression and the properties of MSCs.

### Bioinformatic analysis of integrins alpha *6* gene

A search of the public databases showed eight genes of the human integrin alpha gene family hosting 18 members. In addition, several lncRNA, including three antisense and multiple alternative promoters (Supplementary Table S3). These genes code for exons ranging from 26 to 32, producing 1 to 17 transcripts. These transcripts were generated by promoters ranging from 1–10. *ITGA6* has the most putative promoters. Each of the genes also expressed 0 to 3 lncRNA. One of the lncRNA of *ITGA2*, *ITGA6*, and *ITGA9* was antisense RNA, *ITGA2-AS1, ITGA6-AS1,* and *ITGA9-AS1,* and they are likely to be involved in regulating the respective genes. The identity of 10 promoters of *ITGA6* is shown in Supplementary Table S4. One of *ITGA6* promoters overlaps *ITGA6-AS1* and *ITGA6* exon2 sequences (Figure 1B). The *ITGA6* (NCBI Gene ID: 3,655) occupies a region of 79,124 bps on the forward strand of the short arm of chromosome two mapped to cytogenetic location 2q31.1 at GRCh38/hg38 genomic coordinates: chr2:172,427,336–172,506,459 and generate six mRNA types/variants. The global alignment of two *ITGA6* mRNA variants, *ITGA6* mRNA variant one versus the other five mRNA variants, showed alignment similarities in the range of 91.1%–99.2% (Supplementary Table S5). *ITGA6* mRNA variant one was selected for further bioinformatics analysis in the current study. Thus, we investigated the potential association of *ITGA6-AS1* with *ITGA6* expression, considering the critical roles of *ITGA6* in the determination of MSCs types.

### Potential involvement of integrins alpha *6-*antisense *1* in the regulation of integrins alpha *6*

Since some of the studies have shown that ITGA6 is regulated by ITGA6-AS1 (Song et al., 2021), we investigated the sequence *ITGA6-AS1* transcript pairing with *ITGA6* transcript variant 1. The *ITGA6-AS1* was coded by the opposite strand of DNA coding for the *ITGA6* exon-2 (Figure 1B). Local alignment analysis by EMBOSS Matcher of *ITGA6-AS1* NR_157,573.1 transcript of 442 bp and *ITGA6* transcript type 1 (NM_001079818.3) of 5,686 bp showed partial complementarity with 64.9% similarity along 57 bp (Figure 1C). The identified partial complementarity mapped at positions 5,447 to 5,500 in the 3′untranslated region (UTR) of *ITGA6* mRNA type 1/variant 1 (v1). Furthermore, the *ITGA6-AS1* showed 65.4% alignment to 26 bp of *ITGA6* exon 2 (E2) (Figure 1D).

Knowing the lncRNA loci are sources of microRNA (Sun et al., 2021), we explored the presence of microRNA in lncRNA *ITGA6-AS1* transcript by using miRbase, human (*Homo sapiens*) option tool. Four miRNA sequences identified were referred to by us as human (hsa)-miR-AS1.1, hsa-miR-AS1.2, hsa-miR-AS1.3, and hsa-miR-AS1.4 that showed similarities to hsa-miR-6772–5p, hsa-miR-7109–3p, hsa-miR-6797–5p, and hsa-miR-1911–5p respectively (Figure 1E, and Supplementary Table S6). The in-silico analysis by RNA22 v2 tool for microRNA recognition elements (MRE) in the target transcript showed that miR hsa-miR-AS1.2 could not target *ITGA6* MRE and thus cannot interfere with *ITGA6* transcription. The other three identified miR sequences target *ITGA6* MRE of *ITGA6* transcript at *ITGA6* 3′-UTR and *ITGA6* exon 1 (Supplementary Table S7). The low *p*-values, 6.18E-2–1.21E-1, represent a greater chance of the *ITGA6* transcript containing a valid *ITGA6* MRE.

### Genomic context of integrins alpha *6* exon 2 promoter

**FIGURE 2:**
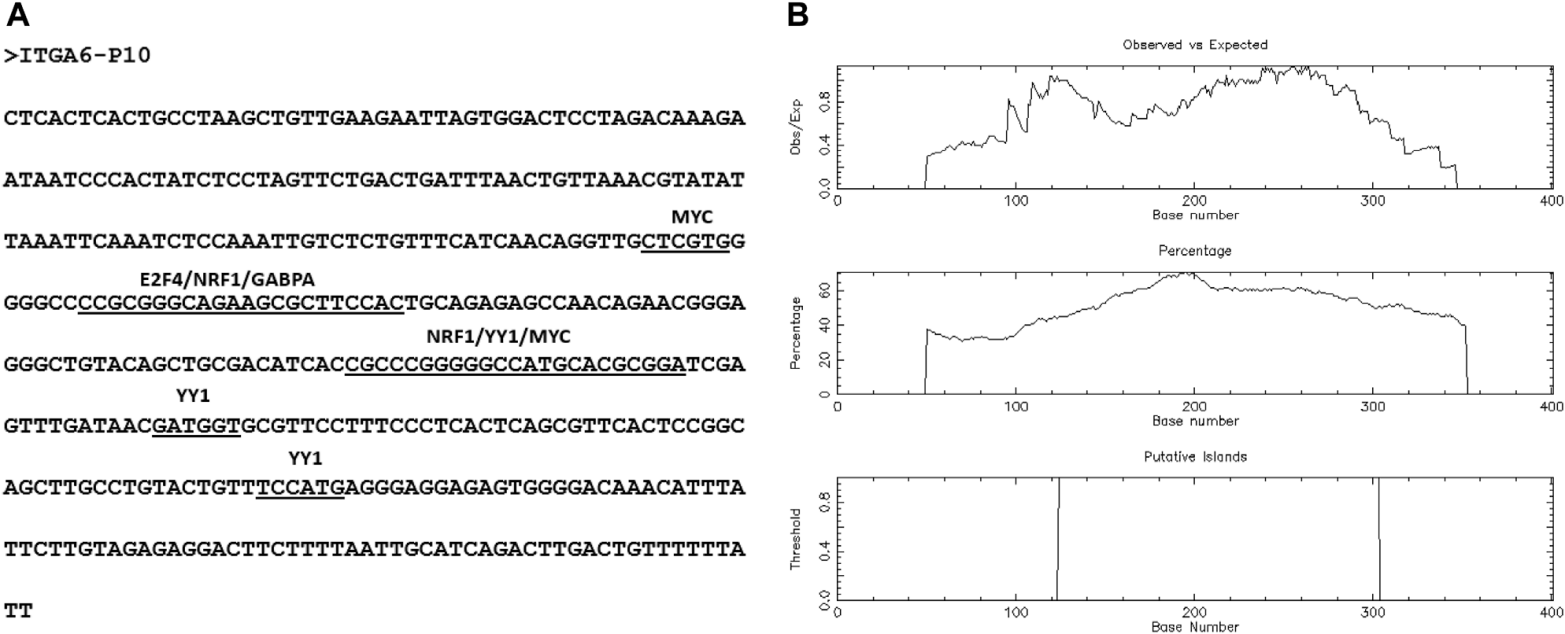
The predicted features of P10, bidirectional promoter. **(A)** P10 sequence shows transcription factors binding sites. **(B)** Identification of CpG Island in the P10 sequence. The following options were searched: Window size, 100; minimum sequence length, 150; minimum Obs/Exp CpG, >0.6; %C + %G, >50.00%.

Ensembl database showed ten *ITGA6* alternative putative promoters from P1 to P10 (Supplementary Table S4). Two of 10 *ITGA6* promoters, P1 and P10, were mapped at *ITGA6* exon-1 and exon-2, respectively, and eight promoters, P2 to P9 mapped in the *ITGA6* intron-1 (Figure 1B). The divergent location of *ITGA6* exon two and *ITGA6-AS1*, overlapping promoter ITGA6 exon-2 (P10) sequences, suggests P10 may possess bidirectional activity. Consistent with this observation, the bioinformatics analysis demonstrated P10 has the features and properties of a bidirectional promoter (Yang and Elnitski, 2008; Seila et al., 2009; Orekhova and Rubtsov, 2013; Al-Obaide et al., 2021). In addition to the divergent configuration of *ITGA6-AS1* and P10, the 402-nucleotide sequence of P10 lacked a TATA box motif and enriched in binding sites for several transcription factors GABPA, MYC, E2F4, NRF1, YY1 found in bidirectional promoters (Figure 2A). Additionally, bioinformatics analysis demonstrated that the *ITGA6*-P10 is CG rich and contains CGI. EMBOSS Cpgplot software revealed a CGI length of 180 bp in the P10 sequence that extends from nucleotide 125 to 304 along P10 sequence (Figure 2B).

### Quantification of expression of integrin alpha *6* and integrin alpha *6-*antisense *1* expression in mesenchymal stem cells

**FIGURE 3:**
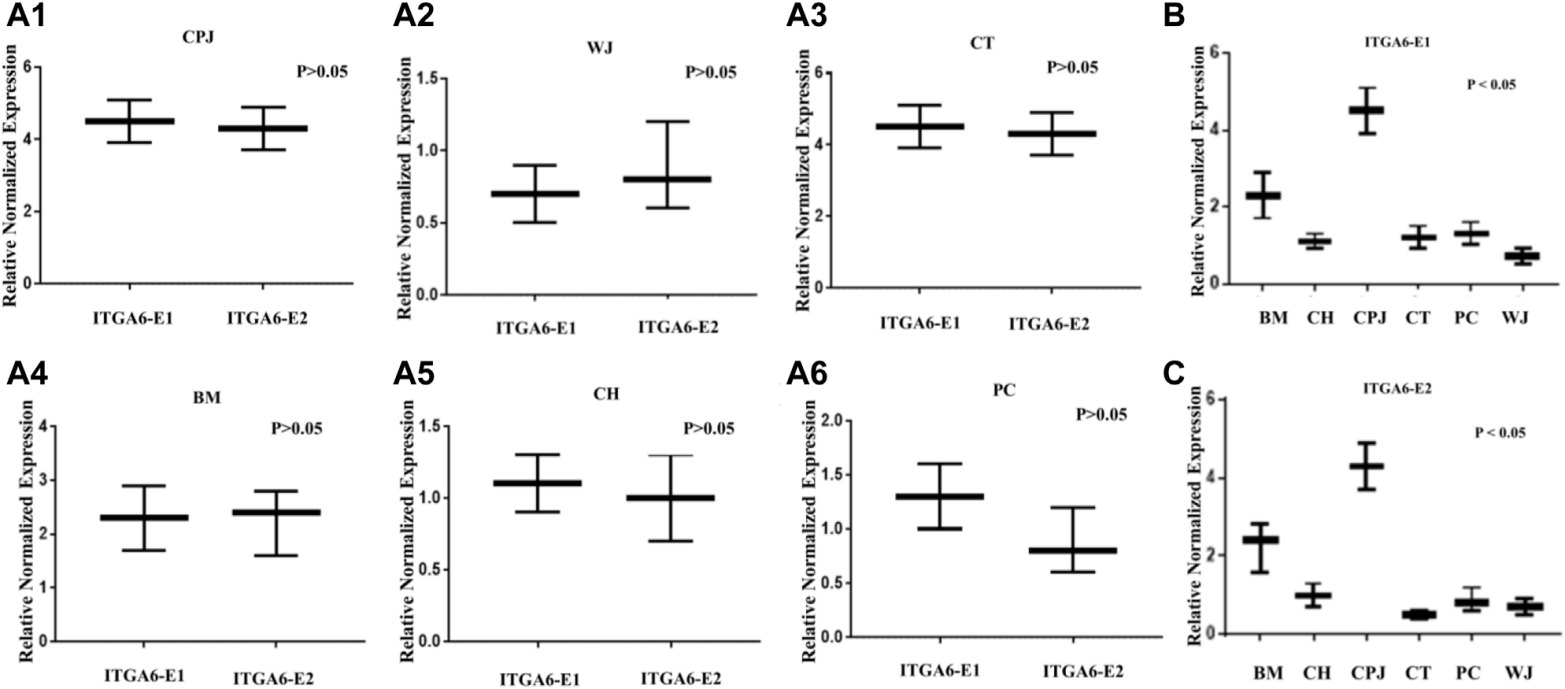
The expression of *ITGA6* in the MSCs. **(A1–A6)** The *t*-test showed no statistically significant difference in expressions of *ITGA6*-E1 and *ITGA6*-E2 in the same MSC, *p* > 0.05. **(B–C)** A one-way ANOVA revealed a statistically significant difference in the expressions of *ITGA6*-E1 and *ITGA6*-E2, respectively in the six MSCs, *p* < 0.05.

To validate the bioinformatic findings and significance that might be influencing the properties of MSCs, we analyzed the expression of both *ITGA6* and *ITGA6-AS1* by RT-qPCR. The results depicted in Figures 3A1–A6 show no significant difference between the expression of *ITGA6*-E1 and *ITGA6*-E2 in the same tested MSCs. However, there was a statistically significant difference in the expressions of *ITGA6*-E1 in the samples of MSCs from various sources (Figure 3B). A similar trend in the expression of *ITGA6*-E2 was observed among various MSCs (Figure 3C). The relative normalized expression values for *ITGA6*-E1 and *ITGA6*-E2 showed that CPJ-MSCs had a 2 to 5-fold increase in expression levels over other MSCs.

**FIGURE 4:**
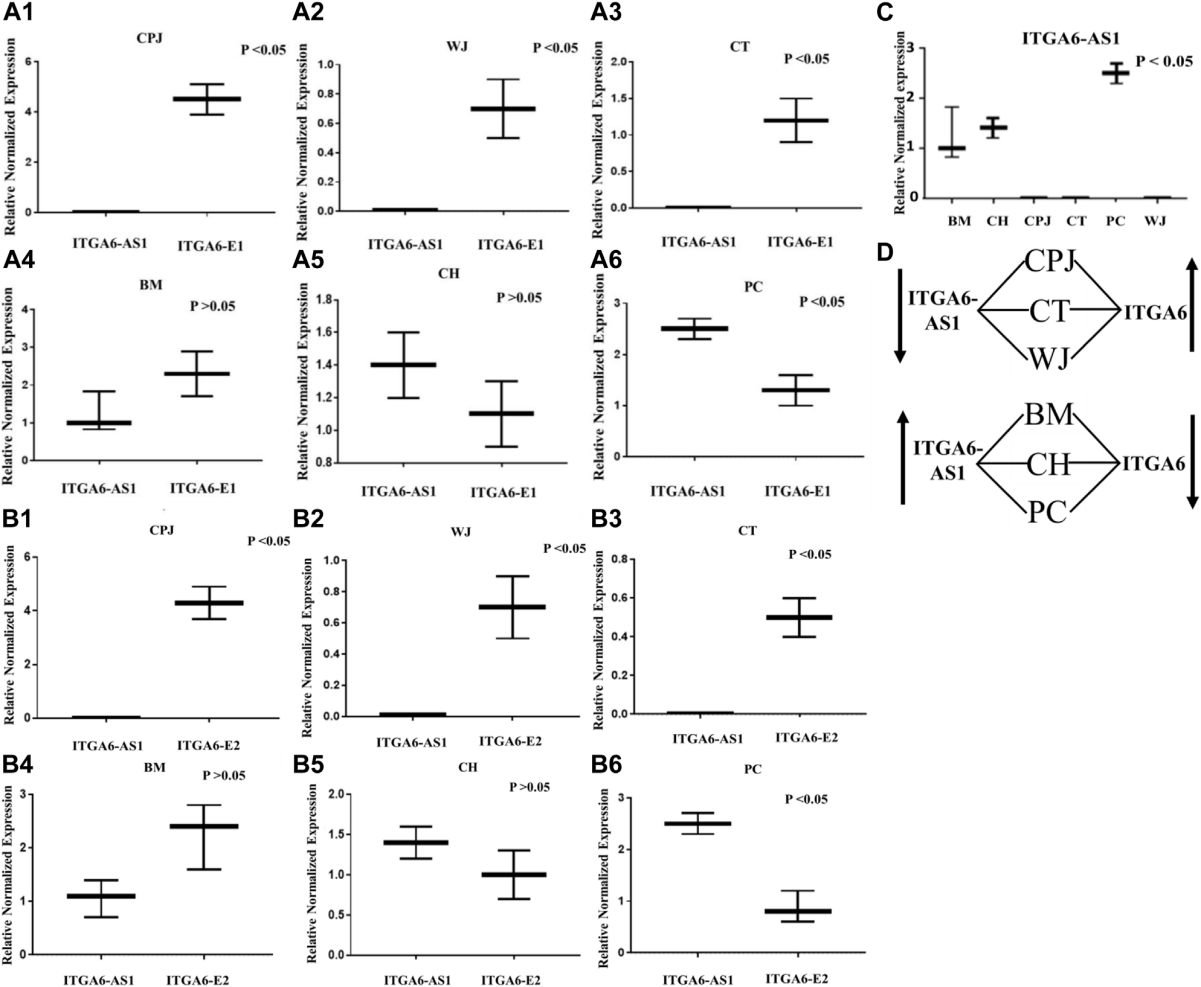
The expression of *ITGA6-E1* and *ITGA6*-*AS1* in MSCs. **(A1–A6)** Expression of *ITGA6-*E1 and *ITGA6-AS1* in the same CPJ, WJ, CT, BM, CH and PC, respectively. **(B1–B6)** Expressions of *ITGA6-*E2 and *ITGA6-AS1* in the same CPJ, WJ, CT, BM, CH and PC, respectively. **(C)** A one-way ANOVA revealed a statistically significant difference in *ITGA6-AS1* expressions in the tested MSCs, *p* < 0.05. **(D)** Sketch showing correlation of *ITGA6-AS1* and *ITGA6* expressions in MSCs.

We then compare the expression of *ITGA6* and *ITGAS6-AS1* in the MSCs from all six sources. The results reported in Figure 4 show that the expression of *ITGA6* was inversely proportional to the *ITGA6-AS1*. The primer set directed against *ITGA6*-E1 indicated significantly higher expression of *ITGA6* but lower expression of *ITGA6-AS1* in MSCs from CPJ, WJ, and CT (Figures 4A1–A3). In contrast, the difference in the expression of *ITGA6* and *ITGA6-AS1* was insignificance in the MSCs from BM and CH (Figures 4A4–A5). On the other hand, the expression of *ITGA6-AS1* was significantly higher than *ITGA6* in PC MSCs (Figure 4A6). A similar trend was noted when the primer set against *ITGA6*-E2 was used (Figures 4B1–B6). The expression of *ITGA6-AS1* was significantly higher in MSCs from BM, CH, and PC when compared to CPJ CT and WJ. The highest expression of the antisense RNA was observed in PC MSCs (Figure 4C). There was a clear pattern in the expression of *ITGA6* and *ITGA6-AS1* in the MSC samples, as summarized in Figure 4D. When *ITGA6-AS1* was high, expression of *ITGA6* was low, suggesting its potential role in the regulation of *ITGA6* in MSCs. Based on the bioinformatics analysis, we proposed that P10 is involved in the expression of *ITGA6-AS1* that overlaps *ITGA6* exon two sequence. However, further studies are warranted to define the function of P10 unambiguously.

## Discussion

ITGA6 is one of the many cell surface markers commonly found in over 30 types of stem cells (Krebsbach and Villa-Diaz, 2017). While ITGA6’s role in a broad range of stem cell populations has been linked to maintaining the self-renewal of pluripotent stem cells and breast and glioblastoma cancer stem cells, its function in the multipotent MSCs is not well understood.

Our study of the six human MSCs provided evidence of a potential correlation between the ITGA6 and its antisense RNA, ITGA6-AS1, and MSCs characteristics for the first time. Elevated expression of *ITGA6* in BM MSCs from different donors was correlated with high clonogenicity, migration, differentiation, low doubling time, and proliferation (Nieto-Nicolau et al., 2020; (Lee et al., 2009; Yu et al., 2012; Nystedt et al., 2013). However, our studies showed low ITGA6 expression, poor clonogenicity, and higher doubling time in BM MSCs. On the other hand, we found significantly higher clonogenicity and ITGA6 expression and low doubling time in MSCs derived from human umbilical cord tissues (CT, CPJ, and WJ).

RNA generated from lncRNA has been shown to be a widespread phenomenon in regulating genes in eukaryotes and humans (Villegas and Zaphiropoulos, 2015). The plausible explanation for the *ITGA6-AS1* regulatory role originates from the transcript’s sequence features. Antisense RNAs are lncRNAs subtypes that can alter the stability and translation of cytoplasmic mRNAs (Statello et al., 2021; Sun et al., 2021). Also, lncRNAs are host for microRNA (miRNA). The miRNA can be produced from non-coding RNA transcripts (Ballantyne et al., 2016; He et al., 2019; Zhang et al., 2019). The three identified miR sequences from *ITGA6-AS1* transcript in this study can potentially target MREs in the *ITGA6* transcript at *ITGA6* 3′-UTR, and *ITGA6* exon 1. Previous studies reported miRNAs could target 5′ and 3′ UTRs as well as exons (Broughton and Pasquinelli, 2016; Plotnikova et al., 2019). The UTRs at the 3′ end of mRNA transcripts play a crucial role in gene expression and contain important sequences that influence the fate of mRNA (Matoulkova et al., 2012; Mayr, 2019). The regulatory mechanism of miRNAs functions through inactivating the target mRNA by the silencing complex (RISC) (Tafrihi and Hasheminasab, 2019; Al-Obaide et al., 2021). Further, in this study, we showed putative partial binding of *ITGA6-AS1* to the complementary region of the *ITGA6* at exon two and 3′UTR, such partial binding may have a consequential post-transcriptional influence on the *ITGA6* mRNA and cause mRNA stabilization or destabilization (He et al., 2019). Although the reported data in this study suggest *ITGA6-AS1* is involved in the *ITGA6* regulation, more detailed studies are required to explore the *ITGA6-AS1* role in *ITGA6* expression in MSCs.

Additional research could be conducted to explore the inverse correlation in the expression of *ITGA6* and *ITGA6-AS1.* The NCBI-Nucleotide and Ensembl databases showed *ITGA6* has several different splice variants; quantifying these variants may indicate transcription preference of *ITGA6* transcript type correlated with *ITGA6-AS1*. Overexpression or inhibition analysis of *ITGA6-AS1* should be performed to assess its effect on *ITGA6* expression. It is also of interest to investigate the interaction between *ITGA6-AS1* and lncRNA–*ITGA6* mRNA transcripts by RNA-RNA pulldown assay. Further work is warranted to show the potential roles of *ITGA6* bidirectional promoter (P10) usage in the expression of *ITGA6-AS1* and *ITGA6* in the MSCs. Our results also detected intrinsic elements in the putative *ITGA6* that generate microRNAs from the *ITGA6-AS1,* which could impart epigenetic regulation of ITGA6. A clear understanding of ITGA6-AS1 regulation of the gene could provide novel ways to improve the therapeutic potential of MSCs.

## Data Availability

The original contributions presented in the study are included in the article/Supplementary Material, further inquiries can be directed to the corresponding author.
