# Supplementary material for: The potential role of integrin alpha 6 in human mesenchymal stem cells
Source: Front Genet. 2022 Sep 16;13:968228. doi: 10.3389/fgene.2022.968228 (PMC9535380; doi:10.3389/fgene.2022.968228)
Supplement: Supplementary file 1 [file Table4.DOCX]

**List of the Contents of this File**
I. Supplementary Tables S1 – S7

1. **Supplementary Tables**

**Table S1.** Designed primers for *ITGA6* exon 1, *ITGA6* exon 2, *ITGA6-AS1* and *GAPDH*.

| **Gene** | **Primers** | **Primers’ Sequence** | **Product Length** |
| --- | --- | --- | --- |
| ITGA6 (Exon 1) | P1 Forward | TGCGAGTCTCCAGAGAACAA (Sense) | 130 |
|  | P1 Reverse | CCACCTTCGCCTCCTCT (Antisense) |  |
| ITGA6 (Exon 2) | P10 Forward | ATCTCCAAATTGTCTCTGTTTCATC (Sense) | 99 |
|  | P10 Reverse | TACAGCCCTCCCGTTCT (Antisense) |  |
| ITGA6-AS1 | AS1-Forward | TTTCCATGAGGGAGGAGAGT (Sense) | 135 |
|  | AS1-Reverse | GCATCCCAGTATGATTCAGAAGTA (Antisense) |  |
| GAPDH | GA- Forward | TCACCAGGGCTGCTTTTAACT (Sense) | 101 |
|  | GA-Reverse | TTCCCGTTCTCAGCCATGTA (Antisense) |  |

**Table S2**. Web-based databases and in-silico tools used in this study.

| **Database/Description** | **URL** |
| --- | --- |
| NCBI-GenBank | https://www.ncbi.nlm.nih.gov/genbank/ |
| Ensembl Genome Browser | https://useast.ensembl.org/index.html |
| UCSC Genome Browser | https://genome.ucsc.edu/index.html |
| EMBL-EBI | https://www.ebi.ac.uk/ |
| Eukaryotic Promoter Database | https://epd.epfl.ch/index.php |
| EMBOSS Matcher | https://www.ebi.ac.uk/Tools/psa/emboss_matcher/ |
| EMBOSS Needle | https://www.ebi.ac.uk/Tools/psa/emboss_needle/ |
| EMBOSS Cpgplot | https://www.ebi.ac.uk/Tools/seqstats/emboss_cpgplot/ |
| miRBase | https://mirbase.org/index.shtml |
| RNA22 | https://cm.jefferson.edu/rna22/ |

**Table S3.** The *ITGA6* gene is one of 18 members of the integrin alpha gene family. All genes mapped at forward strand except five genes *ITGA5, ITGA7, ITGA8, ITGA10, ITGA11* and *ITGAE* mapped at reverse strand. Data from NBCI-Gene and Ensembl databases.

| **Gene name** | **Aliases** | **Gene ID** | | **Map Locations** | **Number of Transcripts** | **Number of Exons** | **Number of Promoters** | | **Number of lncRNA** |
| --- | --- | --- | --- | --- | --- | --- | --- | --- | --- |
| *ITGA1* | VLA1; CD49a | | 3672 | 5q11.2 | 1 | 29 | 2 | 3 | |
| *ITGA2* | BR; GPIa;CD49B; HPA-5;VLA-2; VLAA2 | | 3673 | 5q11.2 | 6 | 30 | 2 | 3  (One antisense) | |
| *ITGA2B* | GT; GT1; GTA; CD41; GP2B; HPA3; CD41B;  GPIIb; BDPLT2; BDPLT16; PPP1R93 | | 3674 | 17q21.31 | 3 | 30 | 3 | - | |
| *ITGA3* | VL3A; CD49C; FRP-2; GAPB3; ILNEB; MSK18; VCA-2; VLA3a; GAP-B3 | | 3675 | 17q21.33 | 3 | 26 | 4 | 2 | |
| *ITGA4* | CD49D, IA4 | | 3676 | 2q31.3 | 2 | 29 | 2 | - | |
| *ITGA5* | CD49e, FNRA, VLA-5, VLA5A | | 3678 | 12q13.13 | 2 | 31 | 2 | - | |
| *ITGA6* | CD49f; VLA-6; ITGA6B | | 3655 | 2q31.1 | 10 | 28 | 10 | 3  (One antisense) | |
| *ITGA7* | - | | 3679 | 12q13.2 | 15 | 33 | 3 | - | |
| *ITGA8* | - | | 8516 | 10p13 | 3 | 31 | 1 | - | |
| *ITGA9* | RLC; ITGA4L; ALPHA-RLC | | 3680 | 3p22.2 | 1 | 28 | 1 | 1  (One antisense) | |
| *ITGA10* | PRO827 | | 8515 | 1q21.1 | 17 | 29 | 2 | - | |
| *ITGA11* | HsT18964 | | 22801 | 15q23 | 3 | 31 | 2 | - | |
| *ITGAD* | ADB2; CD11D | | 3681 | 16p11.2 | 17 | 32 | 3 | 1 | |
| *ITGAE* | CD103;HUMINAE | | 3682 | 17p13.2 | 7 | 31 | 7 | 2 | |
| *ITGAL* | CD11A; LFA-1; LFA1A | | 3683 | 16p11.2 | 7 | 32 | 2 | 2 | |
| *ITGAM* | CR3A; MO1A; CD11B; MAC-1; MAC1A; SLEB6 | | 3684 | 16p11.2 | 7 | 31 | 4 | - | |
| *ITGAV* | CD51; MSK8; VNRA; VTNR | | 3685 | 2q32.1 | 3 | 32 | 1 | - | |
| *ITGAX* | CD11C; SLEB6 | | 3687 | 16p11.2 | 6 | 31 | 4 | - | |

**Table S4**. The *ITGA6* alternative promoters. The EPD promoter (ITGA6_1) and ENSR00000126322 (P1) sequences overlap.

| **Promoter ID** | **Symbol** | **Map Locations at Chromosome 2/+ Strand** | **Span (bp)** |
| --- | --- | --- | --- |
| ENSR00000126322 | P1 | 172,426,600-172,431,801 | 5,202 |
| ENSR00000628172 | P2 | 172,438,000-172,438,601 | 602 |
| ENSR00001040835 | P3 | 172,439,000-172,439,201 | 202 |
| ENSR00000628171 | P4 | 172,440,600-172,440,801 | 202 |
| ENSR00001040838 | P5 | 172,441,000-172,441,401 | 402 |
| ENSR00001040836 | P6 | 172,441,800-172,442,001 | 202 |
| ENSR00001040837 | P7 | 172,442,200-172,442,801 | 602 |
| ENSR00001229442 | P8 | 172,460,600-172,461,001 | 402 |
| ENSR00000628169 | P9 | 172,462,000-172,463,201 | 1,202 |
| ENSR00000628170 | P10 | 172,465,400-172,465,801 | 402 |
| ITGA6_1 |  | 172,426,586-172,427,886 | 1,301 |

**Table S5.** The Global alignment of *ITGA6* mRNA variants. Alignment of *ITGA6* mRNA variant 1 (v1) versus the other five *ITGA6* mRNA variants (v2-v6).

| **Aligned Sequences** | **Length (nt)** | **Similarity** | **Gap(s)** | **Site(s) of the Gap(s)** |
| --- | --- | --- | --- | --- |
| 1: ITGA6-v1  2: ITGA6-v2 | 5686 (v1)  5816 (v2) | 5686/5816 (97.8%) | 130/5816 (2.2%) | v1: 3,327 |
| 1: ITGA6-v1  2: ITGA6-v3 | 5686 (v1)  5551 (v3) | 5339/5863 (91.1%) | 489/5863 (8.3%) | v3: 1  v1: 138  v3: 598  v1: 594  v1: 3,017 |
| 1: ITGA6-v1  2: ITGA6-v4 | 5686 (v1)  5641 (v4) | 5641/5686 (99.2%) | 45/5686 (0.8%) | v4: 2,837 |
| 1: ITGA6-v1  2: ITGA6-v5 | 5686 (v1)  5771 (v5) | 5641/5816 (97.0%) | 175/5816 (3.0%) | v5: 2,837  v1: 3,317 |
| 1: ITGA6-v1  2: ITGA6-v6 | 5686 (v1)  5803 (v6) | 5686/5803 (98.0%) | 117/5803 (2.0%) | v1: 977 |

**Table S6.** Identification of mature miRNA sequence (termed miR) in *ITGA6-AS1 NR_157573.1* transcript of 442 bp. Prediction of the mature miRNA sequence from miRNA/ncRNA transcript by miRBase search tool. Abbreviations: miRNA: MicroRNAs, miR: mature miRNA product, hsa: human miRNA.

| **Name** | **Sequence** | **Similarity** | **Score** | **E-value** |
| --- | --- | --- | --- | --- |
| hsa-miR-AS1.1 | aggcuggagcugagg | hsa-miR-6772-5p | 75 | 2.1 |
|  |  |  |  |  |
|  |  |  |  |  |
|  |  |  |  |  |
|  |  |  |  |  |
| hsa-miR-AS1.2 | caagccacuccuggccaucaag | hsa-miR-7109-3p | 74 | 2.5 |
| hsa-miR-AS1.3 | gaggaaaaggacugggaacacga | hsa-miR-6797-5p | 70 | 5.5 |
|  |  |  |  |  |
| hsa-miR-AS1.4 | auuaccuucaugccuguuggg | hsa-miR-1911-5p | 69 | 6.5 |
|  |  |  |  |  |

**Table S7.** The predicted human miRNA mature sequences (miR) in the *ITGA6-AS1* NR_157573.1 that bind target MREs of *ITGA6* NM_001079818.3 transcript mRNA. The prediction of binding sites of *ITGA6* (NM_001079818.3) microRNA recognition elements (MRE) by RNA22 v2 microRNA target detection tool.

| **Predicted**  **miR** | **Predicted miR Similarity with miRBase Mature miRNA** | **Number of Identified MRE in *ITGA6* mRNA** | **MRE Locations in *ITGA6*** | **Leftmost Position (5’→3’) of Predicted *ITGA6* mRNAs Target Sites** |
| --- | --- | --- | --- | --- |
|  | hsa-miR-6772-5p | 1 | *ITGA6*-E1 | 136 |
| hsa-miR-AS1.1 |  |  |  |  |
|  |  | 3 | *ITGA6* 3’-UTR | 4174, 4335, 5543 |
| hsa-miR-AS1.3 | hsa-miR-6797-5p | 1 | *ITGA6* 3’-UTR | 5077 |
|  |  |  |  |  |
| hsa-miR-AS1.4 | hsa-miR-1911-5p | 2 | *ITGA6* 3’-UTR | 3775, 3960 |
